# Pharmacological Importance of Optically Active Tetrahydro-β-carbolines and Synthetic Approaches to Create the C1 Stereocenter

**DOI:** 10.3390/molecules19021544

**Published:** 2014-01-27

**Authors:** Aino E. Laine, Christopher Lood, Ari M. P. Koskinen

**Affiliations:** Laboratory of Organic Chemistry, Department of Chemistry, School of Chemical Tehcnology, Aalto University, PO Box 16100, Kemistintie 1, Aalto FI-00076, Finland; E-Mails: aino.laine@aalto.fi (A.E.L.); christopher.lood@aalto.fi (C.L.)

**Keywords:** tetrahydro-β-carboline, THβC, pharmacological importance, biological activity, C1-substituted THβC

## Abstract

1,2,3,4-Tetrahydro-β-carbolines (THβCs) are a pharmacologically important group of compounds belonging to the indole alkaloids. C1-Substituted optically active THβCs have been the target of extensive synthetic efforts due to the presence of the scaffold in numerous natural products and synthetic targets. This review briefly summarizes the methods to obtain the C1 stereocenter and concentrates on evaluating the pharmacological importance of optically active C1-substituted THβCs, including their PDE5-inhibitory, antimalarial, antiviral and antitumor activities.

## 1. Introduction

1,2,3,4-Tetrahydro-β-carbolines (THβCs), a compound class within the indole alkaloids, consist of a variety of both simple and complex natural and synthetic compounds [1]. These compounds possess a vast spectrum of biological activities and their use in novel pharmacological applications is under constant study, as the THβC structure is present in drugs currently available on the market, drug candidates under development and many other pharmacologically interesting compounds [2,3,4,5,6,7,8,9,10,11,12,13,14,15,16,17]. 

One synthetically interesting subgroup among the THβCs is the optically active THβCs with C1-substitution. A stereocenter at C1 is a typical feature in natural THβCs and establishment of this stereocenter has received plenty of attention. C1-Substituted THβCs have a wide variety of pharmacological properties, including PDE5-inhibitory [2], antimalarial [3,4,5,6,7,8,9], antiviral [10,11,12,13] and antitumor [14,15,16,17] activities. This review summarizes the methods to create the C1-stereocenter and describes the pharmacological activity of simple C1 substituted THβCs. This review offers a welcome update to a previous review discussing β-carbolines [18]. Furthermore, this is the only review focusing on C1-substituted THβCs and this focus allows covering these compounds in more detail.

## 2. Structure and Occurrence

**Figure 1:**
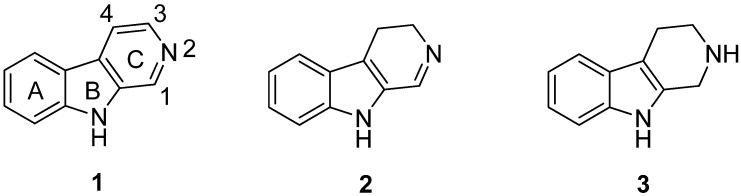
The basic structural units of βC (1), DHβC (2) and THβC (3).

β-Carboline alkaloids are an important group of natural and synthetic indole alkaloids which all bear the common feature of a tricyclic pyrido[3,4-*b*]indole ring structure [19]. The first β-carboline alkaloid recognized was harmalin, originally isolated in 1841 from *Peganum harmala* [20], also known as Syrian rue. The occurrence of β-carbolines in Nature is widespread, presumably due to their simple biogenesis from tryptamine (or tryptophan), and today β-carbolines have been isolated from various plant families, fungi, animal tissues and marine sources [1]. The fully aromatic members of this group are named β-carbolines (βCs) **1**, whereas the members with partially saturated C-rings are known as 3,4-dihydro-β-carbolines (DHβCs) **2** and 1,2,3,4-tetrahydro-β-carbolines (THβCs) **3** (Figure 1). The three rings are referred to as A, B and C-ring, as labeled in structure **1**.

**Figure 2:**
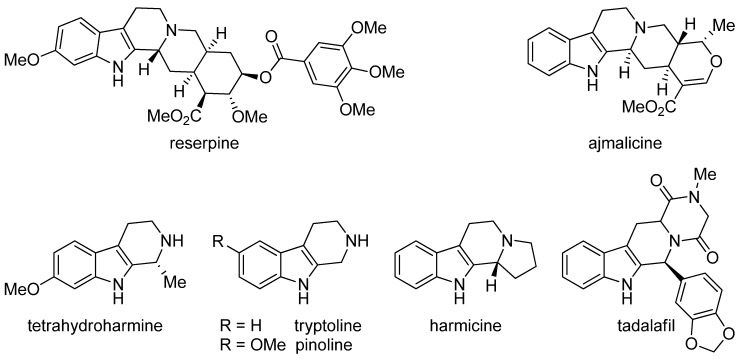
Pharmacologically interesting THβCs.

The best known natural THβCs have been isolated from *Peganum harmala* and *Pausinystalia yohimbe* (formerly *Corynathe yohimbe*). *Yohimbe* alkaloids encompass such pharmacologically interesting natural products as yohimbine and its isomers, reserpine and ajmalicine (Figure 2) the latter two being currently used as antihypertensive drugs. *Harmala* alkaloids include various β-carbolines including the THβCs tetrahydroharmine (an active ingredient in yaje, or ayahuasca, a hallucinogenic brew prepared from the Amazonian plant *Banisteriopsis caapi*), tryptoline, harmicine and pinoline (a melatonin metabolite produced in the pineal gland) [21]. Today, the most important synthetic compound encompassing the THβC structure is tadalafil, which has reached almost $2 billion annual sales in the treatment of erectile dysfunction under the brand name Cialis [2]. Tadalafil is also used for pulmonary arterial hypertension treatment under the brand name Adcirca.

## 3. Biosynthesis

**Scheme 1:**
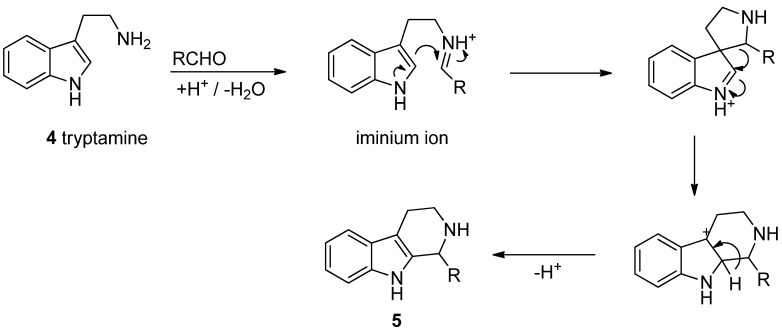
Biosynthesis of THβCs.

The biosynthetic route from tryptamine (**4**) or tryptophan and a carbonyl compound to THβC **5** is simple and the starting materials and their derivatives are widely available in Nature. The reaction from tryptamine to THβC is an enzymatic Pictet-Spengler cyclization and several “Pictet-Spenglerases” have been isolated. The Pictet-Spengler reaction is essentially a two-part reaction (Scheme 1). First, the amine and an aldehyde condense to form an iminium ion. Second, the indole attacks the iminium species from the 3-position, forming a spirocycle that rearranges to a positively charged intermediate which then finally undergoes aromatization via deprotonation to yield the THβC **5** [1,22].

**Figure 3:**
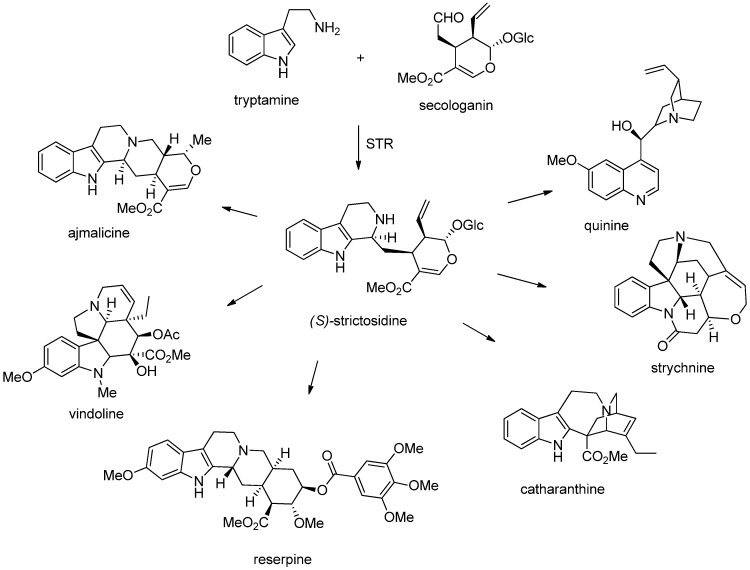
Alkaloids formed from strictosidine.

In the biosynthesis of indole alkaloids, the carbonyl species is often the iridoid glucoside secologanin. The condensation reaction between secologanin and tryptamine is catalyzed by the enzyme strictosidine synthase (STR). The resultant THβC strictosidine is a common precursor for a number of β-carbolines as well as other alkaloids, such as ajmalicine, strychnine, reserpine, quinine, catharanthine and vindoline (Figure 3) [22,23].

## 4. Synthetic Methods to Create the C1 Stereocenter

**Scheme 2:**
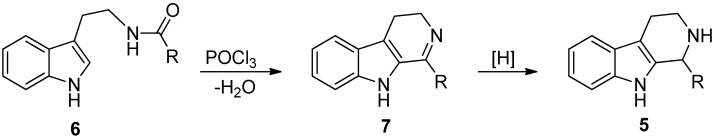
A general Bischler-Napieralski cyclization and reduction to THβC.

The THβC skeleton is found in numerous pharmacologically interesting compounds and hence these alkaloids have been in the focus of synthetic efforts for a long time. The most popular synthetic routes utilize the Pictet-Spengler cyclization [24] (extensively reviewed in 1995 by James Cook [25] and more recently by Joachim Stöckigt in 2011 [23]) that could be considered as a biomimetic approach. Alternatively, a rather similar Bischler-Napieralski cyclization [26] can be used. In a Bischler-Napieralski reaction, a tryptamide **6** is cyclized. Usually dehydration reagents, such as PCl_5_, POCl_3_, SOCl_2_ or ZnCl_2_, are needed to promote the loss of the carbonyl oxygen. The product of the Bischler-Napieralski reaction is a DHβC **7** which can then be further reduced to form the corresponding THβC **5** (Scheme 2). 

**Scheme 3:**
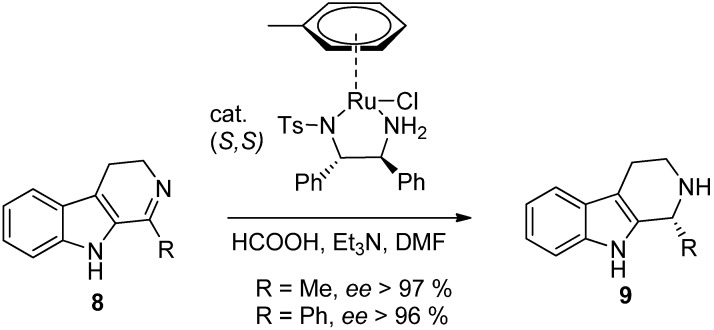
Classical Noyori ATH conditions [27].

Chirality can be introduced to the DHβC product by using asymmetric reduction protocols. Asymmetric transfer hydrogenation (ATH) using Noyori –type catalysts [27] offers a powerful method of accessing a chiral THβC skeleton. Due to the highly stereoselective nature of the reaction in question, this remains one of the most commonly employed procedures. Classical Noyori conditions use an azeotropic mixture of Et_3_N and HCOOH as the hydrogen source to reduce compound **8** to the corresponding chiral THβC **9** (Scheme 3).

**Scheme 4:**
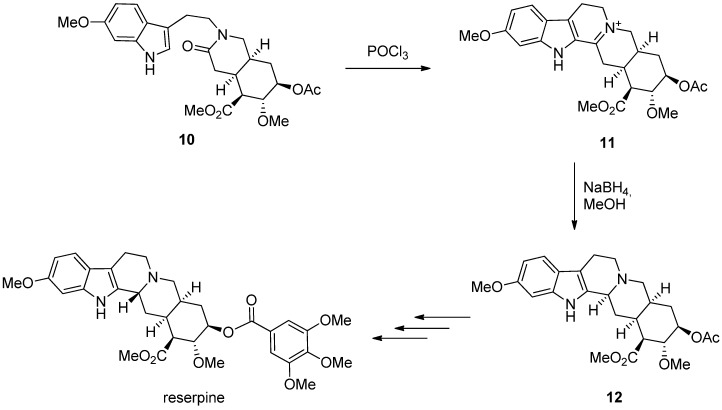
Bischler-Napieralski reaction and diastereoselective reduction in the total synthesis of reserpine [28].

In addition to ATH, the stereochemistry of the reduction product can be controlled also by preexisting directing moieties in a diastereoselective fashion. In Woodward’s classic total synthesis of reserpine [28], published in 1958 (Scheme 4), a Bischler-Napieralski reaction from amide **10** to DHβC **11** was followed by a NaBH_4_ reduction selectively forming THβC **12**. Interestingly but not very surprisingly, this reduction selectively yielded the wrong diastereomer. However, in this case the configuration at C1 could be inverted at a later stage of the synthesis.

**Scheme 5:**
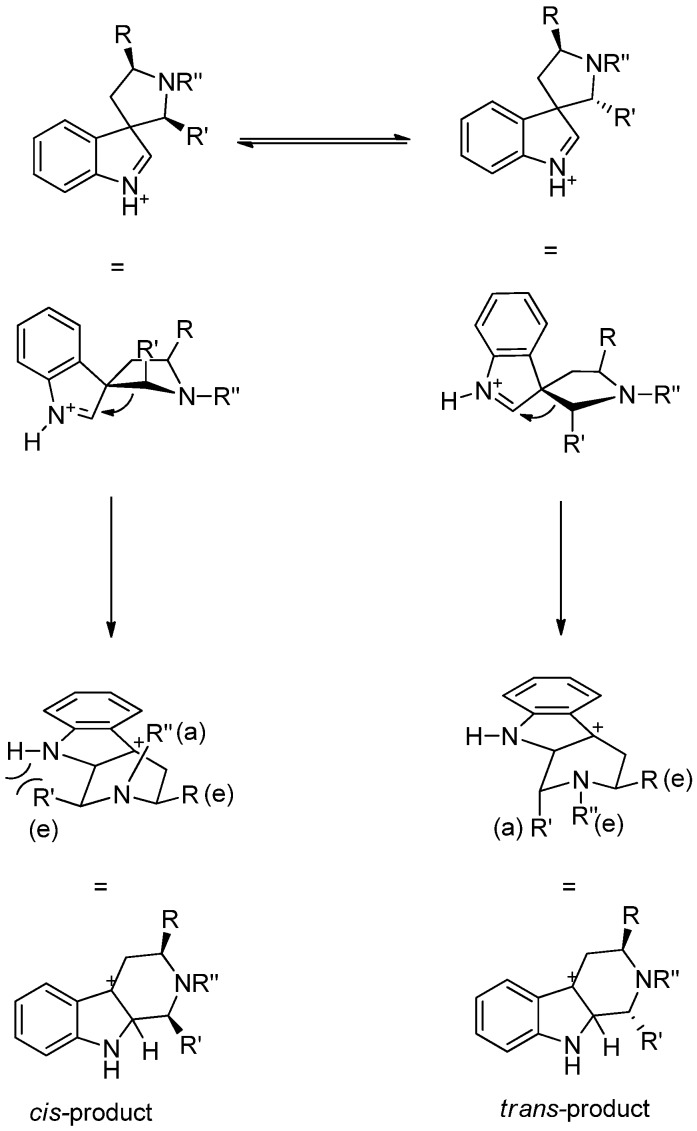
Formation of *cis*- and *trans*-products from the spiroindolenine intermediate. a = axial, e = equatorial.

The stereochemistry in the THβCs can also be controlled by using chiral inductors in the Pictet-Spengler reaction. Internal induction as a means to control the stereochemistry at C1 uses chiral starting materials that are often derived from tryptophan. The existing stereochemistry guides the formation of the second chiral center in cases when C1 is substituted [29,30]. The diastereoselectivity of Pictet-Spengler reaction has been studied and discussed in detail by Bailey and Cook [25,31]. The conformation of the spiroindolenine intermediate determines whether a *trans-* or a *cis*-product is formed (Scheme 5). The *trans*-product is predominantly formed under thermodynamic control and under kinetic control the selectivity is turned towards the *cis*-product. However, the overall control of the *cis/trans-*selectivity is very complicated; in addition to the reaction temperature, the substitution pattern together with the size and electronic properties of the substituents have a considerable impact on the selectivity. 

**Scheme 6:**
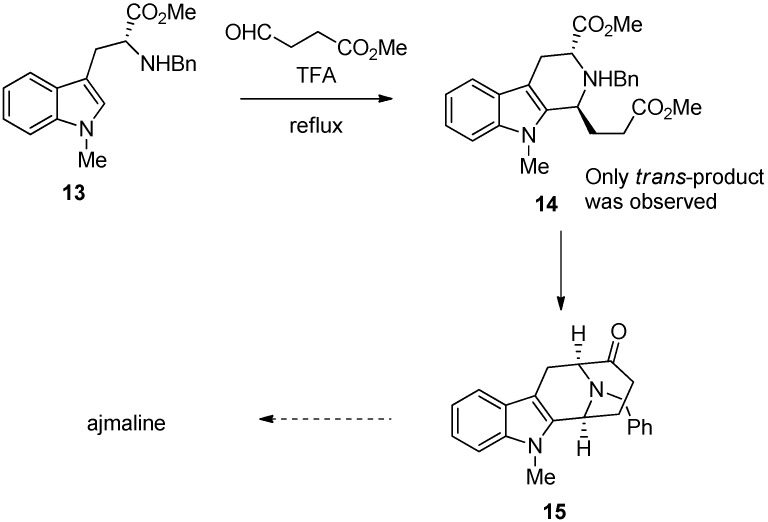
Synthesis of ajmaline by Cook [33].

Despite the complicated nature of this type of internal chiral induction, the reaction outcome has the potential of being highly stereoselective. It has been used extensively in indole alkaloid synthesis to control the stereochemistry at C1 [30,32]. An early example of successful use of internal induction is found in the ajmaline synthesis by Cook (Scheme 6) [33]. In this work, tryptophan benzyl ester **13** was used for the Pictet-Spengler reaction. The yield of the *trans*-product **14** was enhanced by acid induced epimerization that was conducted simultaneously with the Pictet-Spengler reaction.

**Scheme 7:**
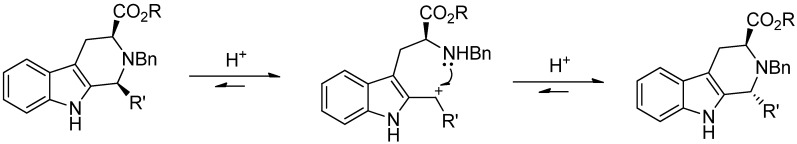
Epimerization of 1,2,3-substituted THβCs favor trans-product.

The key in the epimerization is a reversible ring opening that favors the thermodynamically more stable *trans-*product (Scheme 7). Hence, a reliable protocol exists to yield *trans*-product in very high selectivity from N2 benzyl substituted tryptophan derivatives. The same strategy to reach intermediate **15** has been successfully used to synthetize other related alkaloids such as 11-methoxymacroline and alstophylline [34].

**Scheme 8:**
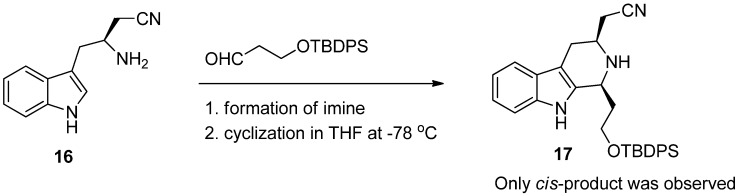
The Kinetically controlled Pictet-Spengler reaction in (−)-raumacline synthesis [35].

Bailey *et al.* have studied kinetically controlled Pictet-Spengler reactions and found that in addition to *trans*-selectivity, under suitable reaction conditions and substitution pattern, the Pictet-Spengler reaction can become highly *cis-*selective [31]. In a representative example (Scheme 8), the cyano substituent in the tryptophan derivative **16** is necessary for the reaction outcome to achieve good *cis*-selectivity, to form product **17**. The kinetically controlled reaction has been subsequently used e.g., in (−)-raumacline synthesis [35] and the conditions leading to the *cis*-selectivity have been studied thereafter [36,37].

**Scheme 9:**
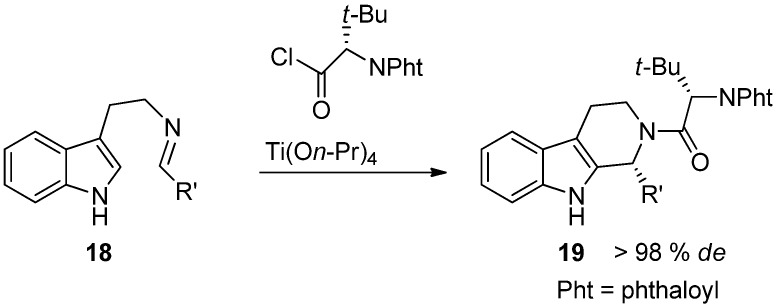
Asymmetric Pictet-Spengler using chiral N2-auxiliary [40].

In addition to a directing group at C3, also chiral auxiliaries on N2 have been studied as an alternative. A benefit of an auxiliary on the nitrogen would be the easy attachment and removal of the chiral auxiliary. However, simple benzyl- or naphthyl-derived chiral groups provide only moderate diastereoselectivity and only 30%–80% *de* [38,39]. Yet, good diastereoselectivities have been obtained using *N,N*-phthaloylamino acids (Scheme 9) [40]. In this example the pre-formed imine **18** is protected with a phthaloylamino acid derivative and the *N*-protected THβC **19** is formed diastereoselectively. 

**Scheme 10:**
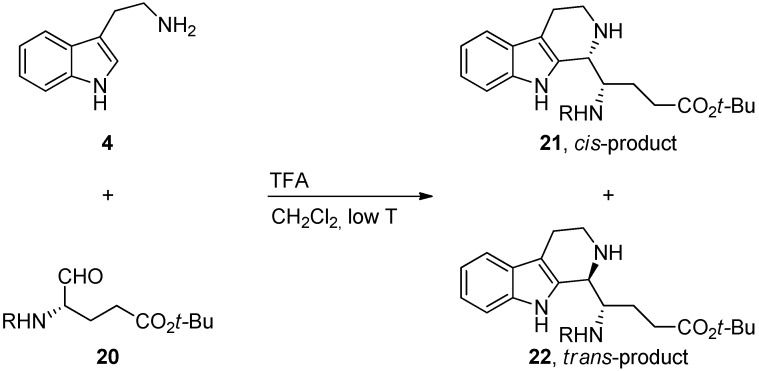
Pictet-Spengler reaction with chiral carbonyl species [41].

Moreover, the source of stereochemical information in Pictet-Spengler reactions can be from chiral carbonyl compounds. Ducrot *et al.* condensed tryptamine **4** with a chiral aldehyde **20** derived from l-glutamic acid (Scheme 10) [41]. The preferred *cis*-compound **21** was formed exclusively when a carboxybenzyl (Cbz) protecting group was used (R = Cbz) and the selectivity was turned towards the *trans*-product **22** when the amine was protected with a pyrrole. Ducrot *et al.* speculated that the size of the protecting group is an important factor, but since pyrrole and Cbz –protecting groups are rather similar in size it seems more likely that this selectivity is guided by other factors. 

**Scheme 11:**
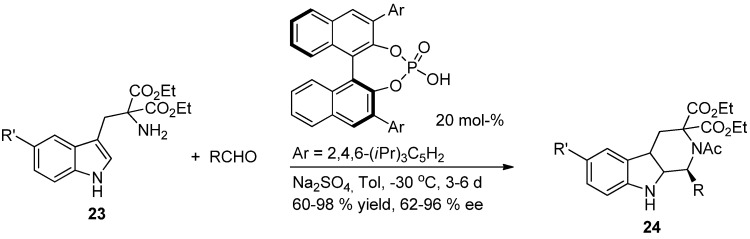
Pictet-Spengler reaction using external asymmetric induction [45].

External asymmetric induction can also be used in the Pictet-Spengler reaction. The first enantioselective Pictet-Spengler reactions using external asymmetric induction were conducted in 1996 by Kawate *et al.* using diisopinocampheylchloroboranes and reaching 90% ee [42]. Today, various asymmetric reagents have been used for Pictet-Spengler reactions providing moderate to high ee:s. In recent publications, popular catalysts in asymmetric Pictet-Spengler reactions includes thiourea based catalysts [43,44] and chiral phosphoric acid diesters [45,46] (Scheme 11). 

Despite the amount of publications related to asymmetric Pictet-Spengler reaction with external asymmetric induction, these methods have several limitations: the C1 substituent usually has to be rather bulky in order to achieve >80% *ee*’s; reaction times can increase to several days and the catalyst loading is often rather high, >10%. 

**Scheme 12:**
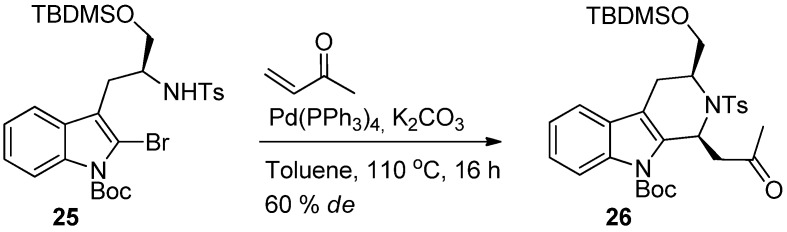
Domino Heck-aza-Michael reaction with asymmetric induction [49].

While Pictet-Spengler and Bichler-Napieralski reactions are the most common methods to build the THβC scaffold, domino reactions incorporating the Heck reaction have also been suggested as a possible approach [47,48]. Recently, Pfeffer *et al.* reported domino Heck-aza-Michael reactions with asymmetric induction [49]. The method provided related N-heterocycles such as tetrahydroisoquinolones with good *de*, however, THβCs were obtained with a modest 60% *de* only (Scheme 12). 

**Scheme 13:**
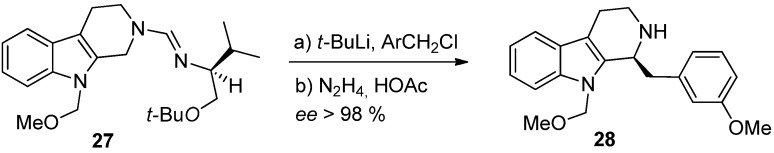
Asymmetric alkylation to C1 [51].

Another example of establishing the C1-stereocenter has been demonstrated by Meyers *et al.* in their total syntheses of (+)-deplancheine and (−)-yohimbine [50,51]. In their work, C1-substitution was introduced at a later stage using the N2-auxiliary as a directing group (Scheme 13). With this method high *ee*’s were obtained. 

## 5. Pharmacological Importance

This chapter concentrates on the pharmacological importance of C1-substituted THβCs. As the skeleton is a common feature in many natural and synthetic compounds, both the multitude of compounds belonging to this group as well as their corresponding biological activities is vast. The review emphasizes recent studies rather than more traditional applications of THβCs. The biochemical and pharmacological functions of β-carbolines (including THβCs) has been reviewed in 2007 [18] as well as the pharmacological importance of indole alkaloid marine natural products in 2005 [52].

### 5.1. Antiprotozoal Activity

**Figure 4:**
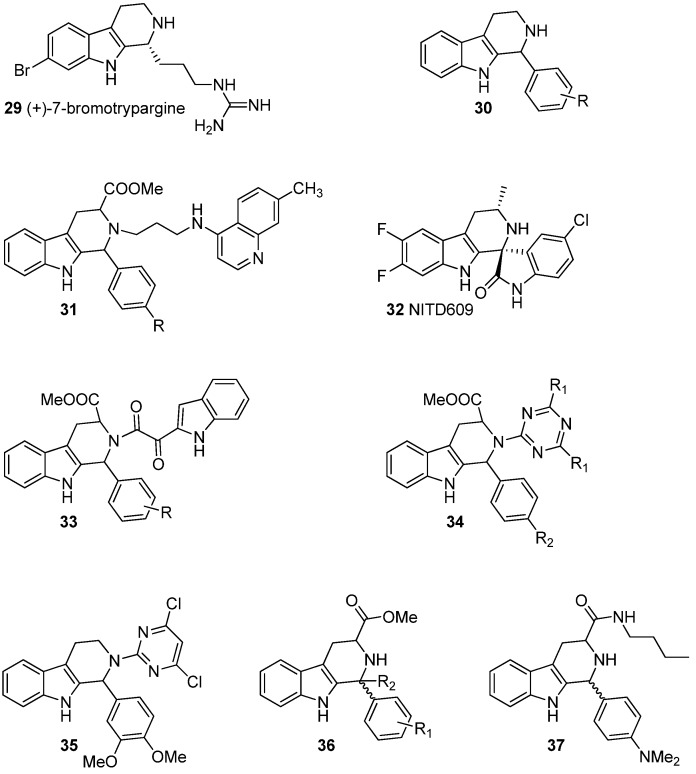
THβCs with antiprotozoan properties.

Several THβCs have been reported to exhibit antiprotozoal, most notably antimalarial, activity (Figure 4). Malaria is one of the most important infectious diseases in the world. According to the World Health Organization (WHO) 200-300 million people are infected and 1.5–2.5 million people die of malaria annually. Some 90% of malaria deaths occur in Africa and 85% of the deceased are younger than 5 years-old [53]. Malaria is caused by red blood cell infecting protozoan parasites belonging to the *Plasmodium* genus, mainly *Plasmodium falciparum* [54]. Traditionally, malaria has been treated with quinine type drugs such as chloroquine. However, the emergence of drug resistant strains has created new challenges for efficient treatments [55]. Several recent studies have focused on the use of different THβC type compounds in the treatment of malaria [3,4,5,6,7,8,9].

(+)-7-Bromotrypargine (**29**) is a marine natural product that was recently isolated from a sponge, *Ancorina* sp. Davis *et al.* reported the isolation and the structural elucidation of the compound together with tests towards antimalarial activity [3]. The compound was tested against both chloroquine-resistant (Dd2) and chloroquine-sensitive (3D7) strains of *P. falciparum* and (+)-7-bromotrypargine was shown to display IC_50_ values of 5.4 μM (Dd2) and 3.5 μM (3D7). Similar compounds were also studied by Chan *et al.* and moderate antimalarial activity was reported [4].

In 2012, Gellis *et al.* synthesized a series of simple 1-substituted THβC derivatives with the general structure **30** with one or more substituents on the phenyl moiety. They tested a series of 20 compounds against the W2 culture adapted strain of *P. falciparum* resistant to chloroquine, pyrimethamine and proguanil and nine compounds showed antiplasmodial activity. The most active compound was a *para-*methoxy-substituted one with IC_50_ of 0.7 µM (W2 IC_50_ of chloroquine 0.7 µM) [5].

In 2008, Gupta *et al.* synthesized a series of chloroquine-THβC hybrid molecules with the general structure **31**. Altogether 23 compounds were screened against chloroquine sensitive *P. falsiparum* strain and the most active compounds had R = *i*-Pr, R = Me and R = Et and showed minimum inhibitory concentrations (MIC) of 0.05, 0.06, and 0.11 μM, respectively, thus showing significantly greater activity than the standard drug chloroquine (MIC = 0.391 μM) [6]. 

A new class of potent antimalarials that has recently gained attention are spiroindolones with a THβC structure. In 2010, these types of compounds were recognized as antimalarials in high-throughput screenings by the Novartis Institute of Tropical Diseases [7,8]. These compounds act against *P. falciparum* with a mechanism distinct from that of the existing antimalarial drugs [7] and the optimized lead compound NITD609 (**32**) has a very high activity of IC_50_ = 0.2 nM [8]. In 2012, NITD609 entered phase 2 clinical trials [9].

In addition to antimalarial studies, THβCs have recently also gained attention as potential antileishmanial and trypanocidal compounds. Leishmaniasis is a tropical infectious disease and the number of people infected with leishmaniasis is ~ 12 million. The annual incidence of leishmaniasis is ~2 million cases and the numbers are increasing. Leishmaniasis is caused by the protozoan flagellate *Leishmania* spp*.*, most notably *L. donovani*, which is spread by sand flies (*Phlebotomus* and *Lutzomyia* spp.). About 90% of leishmaniasis cases occur on the Indian Peninsula, in Brazil and in Sudan [54].

*Trypanosoma* spp. cause trypanosomiasis that can either be manifested as African trypanosomiasis (sleeping sickness) caused by *T. brucei* or as Chagas disease caused by *T. cruzi.* The incidence of African trypanosomiasis is 50,000–70,000 cases annually and it is endemic to the tropical Africa, while Chagas disease occurs in the Middle and Southern America. The approximated number of people with Chagas disease is 8–11 million [54]. 

It has been known for a long time that such complicated THβC alkaloids as α-yohimbine, corynanthine and buchtienine exhibit antileishmanial activity [56,57]. However, during the last 5 years a new interest has arisen towards smaller, synthetic THβC derivatives and several publications have reported antileishmanial activity. In 2010, Chauhan *et al.* synthesized a series of indolylglyoxylamides with the general structure **33** and reported good antileishmanial activities with IC_50_ values of 3.79 µM and 5.17 µM for the *ortho-*bromosubstituted and *para-*ethylated compounds, respectively [58]. These values were several folds better than the standard drug activities (IC_50_ of pentamidine: 20.43 µM). Kumar *et al.* have reported triazine derivatives **34** as well as other similar derivatives **35** as leishmanicidals [59,60]. The triazino derivatives have also been tested *in vivo*. Gellis *et al.* have tested their antimalarial compounds with the general structure **30** for antileishmanial activity. A *p-*bromosubstituted compound showed the most promising inhibitory activity towards *L. Donovani,* with IC_50_ value of 6.1 µM (IC_50_ of pentamidine: 6.3 µM) [5].

Some THβC derivatives have also been studied for trypanocidal activity. In 2010, Tonin and Valdez published studies on similar THβC derivatives (**36** and **37**) [61]. These compounds showed promising activity and compound **37** has been further studied for synergistic activity with other medication [62] but these publications remain the only publications so far on trypanocidal activity of THβC derivatives.

### 5.2. Antiviral Activity

**Figure 5:**
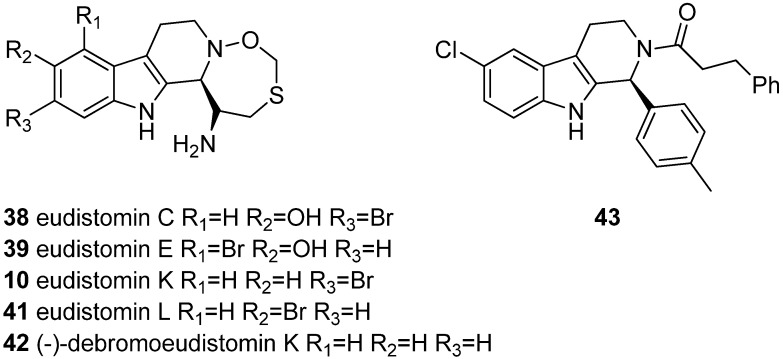
THβCs with antiviral properties.

THβCs have been recognized as antiviral compounds since 1984 when Rinehart *et al.* first studied eudistomins against herpes simplex virus-1 (HSV-1). Eudistomins are marine alkaloids isolated from the colonial tunicate *Eudistoma olivaceum*, and four eudistomins contain the THβC scaffold (**38**–**41**, Figure 5) [10,11].

In addition to the basic THβC structure, eudistomins C, E, K, and L have a condensed oxathiazepine ring system, only reported in these compounds. It has been reported that these four eudistomins have *in vitro* activities against Herpes simplex virus-1 (HSV-1) ranging from 25–250 ng/12.5 mm disc [10]. Later it was also reported that eudistomin K showed activity against the polio vaccine type-1 virus [63]. Eudistomins C and E are also known to possess activities against RNA viruses such as Coxsachie A-21 virus and equine rhinovirus [11]. In 1992, (−)-debromoeudistomin K (**42**) and its structural analogues were tested against a number of viruses and significant antiviral activities were reported against influenza A and B in Madin-Darby canine kidney (MDCK) cells. Activities have been reported also against respiratory cyncytial virus, vesicular stomatitis virus, Coxsachie virus B4 and polio vaccine type-1 virus [12].

The antiviral activities of these eudistomins have never been further studied or developed, but a different series of THβCs have been more recently studied against the human papilloma virus (HPV). In a study at GlaxoSmithKline, a series of 1-substituted THβC derivatives were optimized and resulted in compound **43** possessing nanomolar activity against HPV. The optimized compound had an activity of IC_50_ = 23 nm [13]. GlaxoSmithKline has patented the use of this type of THβCs for the treatment of HPV [64].

### 5.3. Anticancer

Since the 1980s, THβC derivatives have been tested against cancer cell lines. During the last decade, the interest has increased tremendously as traditional THβC targets such as the mitotic kinase Eg5 and phosphodiesterase 5 have been recognized as cancer targets. 

**Figure 6:**
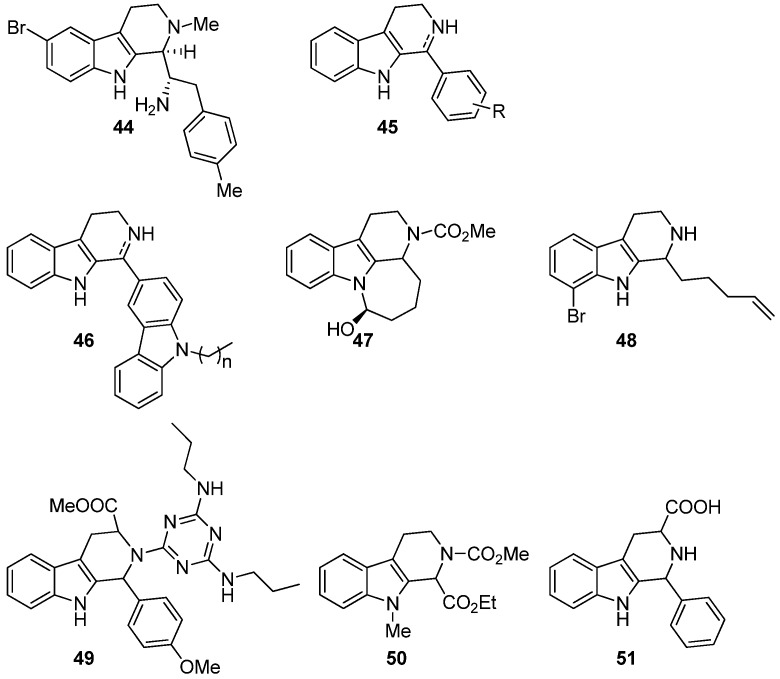
THβCs with cytotoxicity activity.

The first reports on the cytotoxicity of compounds with THβC structure came in 1990 when the newly isolated eudistomins where studied for antileukemic properties. Eudistomin B (**44**, Figure 6) showed antitumor activity against leukemic cell lines L1210 and L5178Y [65]. Later also eudistomin K (**40**, Figure 5**)** was described as an antitumor lead against the murine leukemia cell line P-388, the human leukemia cell line L-1210 and human adenocarcinoma cell lines A-549 and HCT-8 [12]. Eudistomin E (**39**) is also active against the human mouth epidermal carcinoma KB cell line [66]. Apart from eudistomins, few THβC derivatives had been studied for antitumor properties until recent years. However, the group of THβCs that are today recognized as antitumor compounds is growing.

In 2005, Shen *et al.* synthesized a series of simple THβC and DHβC derivatives with the general structure **45**. The compounds were examined against the murine cell line P-388 and the human cell lines KB-16 and A-549, and the human colon adenocarcinoma cell line HT-29. All synthesized compounds exhibited moderate cytotoxicity [67]. In 2011, Shen *et al.* published a new study in which they had increased the size of the substituent in C1 and had a series of THβCs and DHβCs with general structure **46**. The series was evaluated for antitumor activity against human tumor cells including KB, DLD, NCI-H661, Hepa, and HepG2/A2 cell lines. In this study, the DHβC derivatives gave generally better results though also the THβCs showed significant cytotoxicity [14]. 

In 2009, Santos *et al.*, inspired by arborescidine alkaloids, synthesized tetracyclic compounds resembling arborescidines and tested them for antitumor activity towards human lung fibroblasts (MRC-5), human gastric adenocarcinoma (AGS), human lung cancer (SK-MES-1), human bladder carcinoma (J82) and human leukemia (HL-60) cells [68]. From the arborescidine resembling compounds, compound **47** showed most activity having IC_50_ values in micromolar range. The research group also tested all the intermediate compounds they had synthesized and found that the non-cyclic compound **48** actually gave better response to almost all tested cell lines with IC_50_ values ranging from 8.8 to 18.1 µM for lung fibroblasts, gastric adenocarcinoma, lung cancer and bladder carcinoma (IC_50_ of standard etoposide: 0.36–3.93 µM). Kumar *et al.* have also tested their leishmanicidal triazine THβC hybrids (**34**, Figure 4) for cytotoxicity and found that they display nanomolar cytotoxic activity. Their best hit was compound **49**, which had an IC_50_ value of 122 nM [69]. In 2012, Skouta *et al.* synthesized a series of 1,2-disubstituted THβCs and found that compound **50** showed a unique selectivity towards tumorigenic *versus* non-tumorigenic cells and induced cell death without the activation of caspases, hence inducing a *non-apoptotic* cell death [15]. Simple 1,3-disubstituted THβCs have also been tested for cytotoxic activity against the insect origin *Spodoptera frugiperda* Sf9 cell line and the most promising compound was 1-phenyl-THβC-3-carboxylic acid (**51**). Furthermore, these compounds experienced substantial insecticidal activity against mosquito larvae of *Culex pipiens quinquefasciatus* species and mustard aphid (*Lipaphis erysimi*) [70].

**Figure 7:**
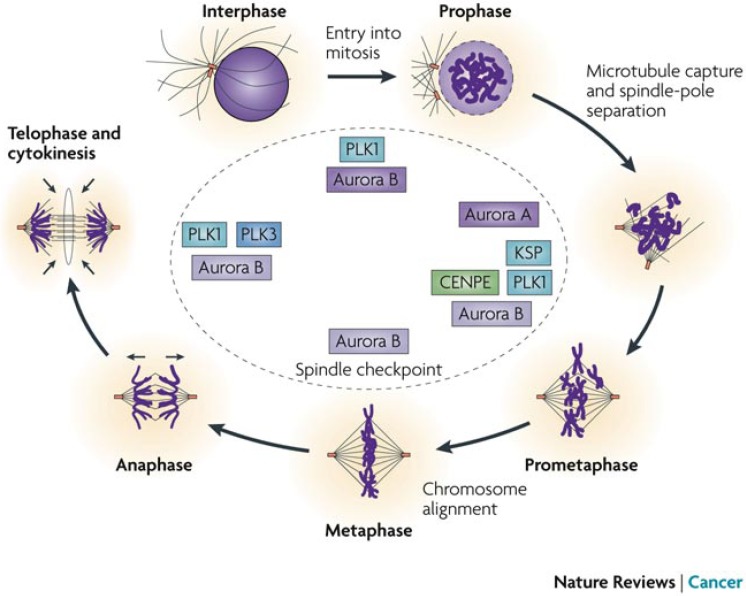
Mitosis and the mitotic kinesins involved in the five steps [72].

Today, one very interesting feature in THβCs is their recognition as mitotic kinesin spindle protein (KSP, also referred to as Eg5) inhibitors. Mitosis is the part of cell division in which the chromosomes condense and divide into two identical sets. The mitotic kinesins are intimately involved in the formation of the mitotic spindle, chromosome segregation, checkpoint control and cytokinesis (Figure 7). The kinesin spindle proteins are highly expressed in breast, ovary, colon, lung, uterine and retinoblastoma tumors [71].

**Figure 8:**
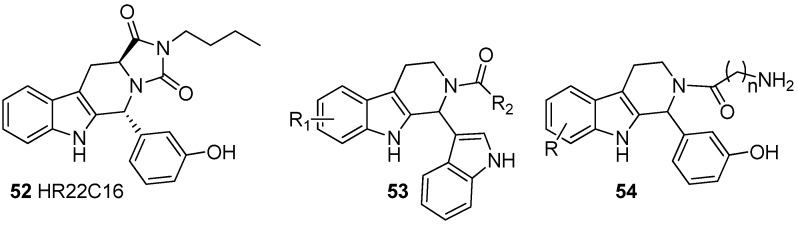
Mitotic kinesin spindle protein (KSP) inhibitors.

KSPs became an important cancer target when monastrol, the first KSP inhibitor, was discovered in 1999. During the last decade, the development of KSP inhibitors has been rapid and many pharmaceutical companies now have KSP inhibitor drugs in clinical trials [71]. During the last ten years, several papers have been published on the KSP inhibitory properties of THβC derivatives (Figure 8) [16,73,74,75,76,77,78]. The mitotic kinesin spindle proteins as cancer targets have been the subject of several recent reviews such as the extensive reviews by Schmidt and Bastians in 2007 [79] and Chan *et al.* in 2012 [80].

In 2003 Hotha *et al.* published the results of an extensive screening that revealed a THβC derivative HR22C16 (**52**) as a potential lead compound for KSP inhibition. They reported that HR22C16 had an IC_50_ value of 800 nM against KSP [16]. After the discovery of HR22C16, several related derivatives and their inhibitory actions have been reported. In 2005, Sunder-Plassmann *et al.* published a series of HR22C16 derivatives and reported that replacing the N-butyl side chain with an N-benzyl side chain increases inhibitory activity to IC_50_ = 650 nM [73]. These type of indolopyridines were patented in 2009 as KSP inhibitors by a German pharmaceutical company, 4SC [74]. The company has now one KSP inhibitor in clinical Phase I trials (SC4-205) [75] and although its structure is not yet revealed, it was speculated in a recent review that it is based on the indolopyridine scaffold [76].

**Figure 9:**
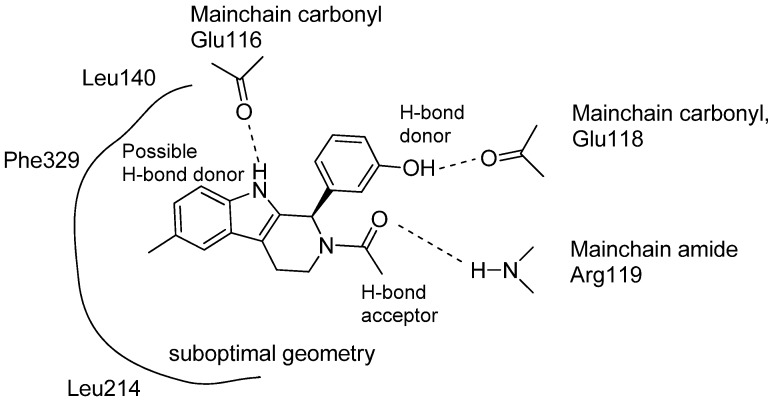
Schematic presentation of interactions between the ATP binding pocket of KSP and inhibitor [78].

HR22C16 inspired compounds have also been further studied by Liu *et al.* who reported that the metabolically liable phenol group can be replaced with indolyl without losing inhibitory activity [77]. The research group also replaced the fourth ring in the HR22C16 structure with a simple acyl group on N2 giving compounds of general structure **53**, thus returning to the original THβC three-ring system. Barsanti *et al.* also published a paper in which 1,2-disubstituted THβCs **54** were evaluated as KSP inhibitors [78]. The structures of Barsanti’s compounds **54** and Liu’s compounds **53** are highly similar. Barsanti’s most promising lead had an IC_50_ value of 58 nM. The group was also able to co-crystallize the inhibitor with KSP making it possible to observe the major interactions in the binding site of KSP with their ligand (Figure 9).

A novel application for THβCs arose when phosphodiesterase 5 (PDE5) became a promising cancer treatment target. Phosphodiesterases are enzymes that catalyze the breakdown of cyclic guanosine monophosphate (cGMP) to guanosine monophosphate (GMP). PDE5 inhibition is one of the common targets for compounds with the THβC structure. It has traditionally been a target for treating erectile dysfunction and pulmonary arterial hypertension. Increased concentration of cGMP in vascular smooth muscle cells leads to vasodilation and subsequently erection. In 2006, Serafini *et al.* first recognized PDE5 inhibitors as antitumor agents [17]. The use of PDE5 inhibitors in the treatment of cancer was reviewed in 2009 [81].

**Figure 10:**
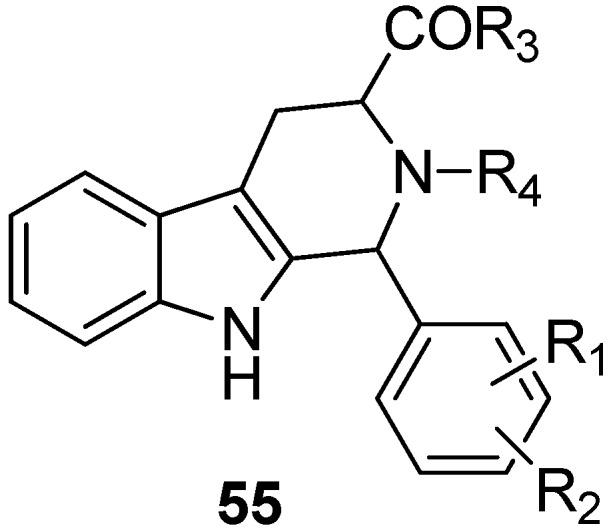
PDE5 inhibitor.

As tadalafil (Figure 2) was one of the PDE5 inhibitors Serafini *et al.* used when testing antitumor properties, it is not surprising that many papers discuss the cytotoxicity of tadalafil-inspired compounds. Tadalafil acts as a PDE5 inhibitor in low nanomolar range and analogues with similar IC_50_ values have been synthesized [82,83,84,85]. The generalized Markush structure **55** (Figure 10) and its use as a PDE5 inhibitor was patented in 2011 [86].

### 5.4. Other Pharmacological Uses

Complex natural alkaloids that contain the THβC structure such as yohimbine or reserpine have a wide range of pharmacological activities. The extracts from *Rauwolfia* spp. has been a part of traditional medicine in tropical and subtropical areas. Some known mechanisms of action of this type of molecules are serotonin receptor (5HT) antagonism and α-adrenergic receptor antagonism. Hence these molecules have a profound effect on the CNS, being hallucinogens, vasodilators and analgesics. However, as the range of activities is broad and these compounds lack inherent selectivity, they haven’t been very useful in modern medicine [87]. 

**Figure 11:**
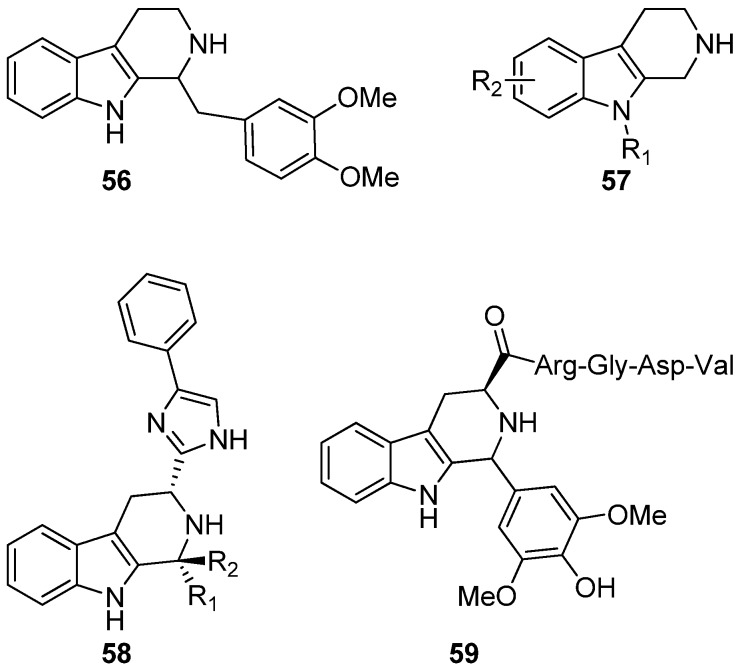
THβC derivatives with miscellaneous pharmacological activities.

Serotonin receptor antagonism has been studied with simple THβCs. An example of such a study was done in the Lilly research laboratories in 1996 by Audia *et al.* who synthetized a series of 1-substituted THβCs in which the substituent consisted of various benzyl or naphthyl groups, as in compound **56** (Figure 11) [88]. The compounds showed moderate selective antagonism towards the 5HT_2B_–receptor. Similar studies were conducted by Giorgioni *et al.* in 2005 [89]. No recent studies have been published on THβCs as 5HT antagonists.

During the last decade, several novel receptor interactions and possible applications of THβC derived compounds have been suggested. Glennon *et al.* has reported the binding of simple C1-unsubstituted THβCs **57** to imidazole receptors I_2_ and I_3_ [90]. In 2001, Poitout *et al.* first described 1,3-substituted THβC derivatives **58** as selective somatostatin receptor type 3 (SSTR3) antagonists [91,92]. Somatostatin receptors are G-protein coupled receptors inhibiting adenylyl cyclase, thus exerting various other effects on intracellular messenger systems. SSTRs are known to mediate cognitive effects, growth hormone inhibition and insulin secretion inhibition [93]. Merck and Co have been granted a patent in the use of THβC based compounds similar to **58** as SSTR3 antagonists in the treatment of type 2 diabetes mellitus [94]. 

THβC derivatives have also been patented for several other uses: protein tyrosine phosphatase (PTP) inhibition [95], growth hormone secretagogue receptor (GHSR) antagonism [96] and histamine receptor modulation [97]. In 2013, THβC derivatives were reported to target fatty acid amide hydrolase (FAAH) and transient receptor potential (TRP) channels [98]. Furthermore, two publications have suggested that THβC RGD peptidomimetic conjugate **59** acts as an antithrombotic agents and have free radical scavenging properties [99,100].

## 6. Conclusions

This review illustrates the pharmacological importance of C1-substituted optically active THβCs featuring numerous recent studies. Development has been rapid especially in antitumor applications as well as in antimalarial applications. Moreover, several novel targets have been recently recognized. Although methods to establish the C1 stereocenter exist, there is room for development and additional contributions. 

## References

[1] Hesse M. (2002). Alkaloids: Nature’s Curse or Blessing.

[2] Daugan A., Grondin P., Ruault C., le Monnier D.G., Coste H., Kirilovsky J., Hyafil F., Labaudiniere R. (2003). The discovery of tadalafil: A novel and highly selective PDE5 inhibitor. 1: 5,6,11,11a-Tetrahydro-1H-imidazo[1',5':1,6]pyrido[3,4-B]indole-1,3(2H)-dione analogues. J. Med. Chem..

[3] Davis R.A., Duffy S., Avery V.M., Camp D., Hooper J.N.A., Quinn R.J. (2010). (+)-7-Bromotrypargine: An Antimalarial β-Carboline from the Australian Marine Sponge *Ancorina* Sp.. Tetrahedron Lett..

[4] Chan S.T.S., Pearce A.N., Page M.J., Kaiser M., Copp B.R. (2011). Antimalarial β-Carbolines from the New Zealand Ascidian *Pseudodistoma opacum*. J. Nat. Prod..

[5] Gellis A., Dumètre A., Lanzada G., Hutter S., Ollivier E., Vanelle P., Azas N. (2012). Preparation and antiprotozoal evaluation of promising β-carboline alkaloids. Biomed. Pharmacother..

[6] Gupta L., Srivastava K., Singh S., Puri S.K., Chauhan P.M.S. (2008). Synthesis of 2-[3-(7-Chloro-Quinolin-4-ylamino)-Alkyl]-1-(Substituted Phenyl)-2,3,4,9-Tetrahydro-1H-β-Carbolines as a New Class of Antimalarial Agents. Bioorg. Med. Chem. Lett..

[7] Rottmann M., McNamara C., Yeung B.K.S., Lee M.C.S., Zou B., Russell B., Seitz P., Plouffe D.M., Dharia N.V., Tan J. (2010). Spiroindolones, a potent compound class for the treatment of malaria. Science.

[8] Yeung B.K.S., Zou B., Rottmann M., Lakshminarayana S.B., Ang S.H., Leong S.Y., Tan J., Wong J., Keller-Maerki S., Fischli C. (2010). Spirotetrahydro β-carbolines (spiroindolones): A new class of potent and orally efficacious compounds for the treatment of Malaria. J. Med. Chem..

[9] IFPMA Developing World Health Partnerships: Novartis R&D for Malaria.

[10] Rinehart K.L., Kobayashi J., Harbour G.C., Hughes R.G., Mizsak S.A., Scahill T.A. (1984). Eudistomins C, E, K, and L, potent antiviral compounds containing a novel oxathiazepine ring from the caribbean tunicate *Eudistoma olivaceum*. J. Am. Chem. Soc..

[11] Rinehart K.L., Kobayashi J., Harbour G.C., Gilmore J., Mascal M., Holt T.G., Shield L.S., Lafargue F. (1987). Eudistomins A-Q, β-Carbolines from the Antiviral Caribbean Tunicate *Eudistoma. olivaceum*. J. Am. Chem. Soc..

[12] Van Maarseveen J.H., Hermkens P.H.H., de Clercq E., Balzarini J., Scheeren H.W., Kruse C.G. (1992). Antiviral and antitumor structure-activity relationship studies on tetracyclic eudistomines. J. Med. Chem..

[13] Miller J.F., Turner E.M., Sherrill R.G., Gudmundsson K., Spaltenstein A., Sethna P., Brown K.W., Harvey R., Romines K.R., Golden P. (2010). Substituted tetrahydro-β-carbolines as potential agents for the treatment of human papillomavirus infection. Bioorg. Med. Chem. Lett..

[14] Shen Y., Chang Y., Lin C., Liaw C., Kuo Y.H., Tu L., Yeh S.F., Chern J. (2011). Synthesis of 1-substituted carbazolyl-1,2,3,4-tetrahydro- and carbazolyl-3,4-dihydro-î²-carboline analogs as potential antitumor agents. Mar. Drugs.

[15] Skouta R., Hayano M., Shimada K., Stockwell B.R. (2012). Design and synthesis of pictet–spengler condensation products that exhibit oncogenic-ras synthetic lethality and induce non-apoptotic cell death. Bioorg. Med. Chem. Lett..

[16] Hotha S., Yarrow J.C., Yang J.G., Garrett S., Renduchintala K.V., Mayer T.U., Kapoor T.M. (2003). HR22C16: A potent small-molecule probe for the dynamics of cell division. Angew. Chem..

[17] Serafini P., Meckel K., Kelso M., Noonan K., Califano J., Koch W., Dolcetti L., Bronte V., Borrello I. (2006). Phosphodiesterase-5 inhibition augments endogenous antitumor immunity by reducing myeloid-derived suppressor cell function. J. Exp. Med..

[18] Cao R., Peng W., Wang Z., Xu A. (2007). Carboline alkaloids: Biochemical and pharmacological functions. Curr. Med. Chem..

[19] Allen J.R.F., Holmstedt B.R. (1980). The simple β-carboline alkaloids. Phytochemistry.

[20] Goebel F. (1841). Über das Harmalin. Justus Liebigs. Ann. Chem..

[21] Sumpter W.C., Miller F.M. (1954). Heterocyclic Compounds with Indole and Carbazole Systems.

[22] Maresh J.J., Giddings L., Friedrich A., Loris E.A., Panjikar S., Trout B.L., Stöckigt J., Peters B., O’Connor S.E. (2008). Strictosidine synthase: Mechanism of a pictet-spengler catalyzing enzyme. J. Am. Chem. Soc..

[23] Stöckigt J., Antonchick A.P., Wu F., Waldmann H. (2011). The pictet-spengler reaction in nature and in organic chemistry. Angew. Chem. Int. Ed..

[24] Pictet A., Spengler T. (1911). Über die bildung von isochinolin-derivaten durch einwirkung von methylal auf phenyl-äthylamin, phenyl-alanin und tyrosin. Ber. Deutsch. Chem. Gesell..

[25] Cox E.D., Cook J.M. (1995). The pictet-spengler condensation: A new direction for an old reaction. Chem. Rev..

[26] Bischler A., Napieralski B. (1893). Zur kenntniss einer neuen isochinolinsynthese. Ber. Deutsch. Chem. Gesell..

[27] Uematsu N., Fujii A., Hashiguchi S., Ikariya T., Noyori R. (1996). Asymmetric transfer hydrogenation of imines. J. Am. Chem. Soc..

[28] Woodward R.B., Bader F.E., Bickel H., Frey A.J., Kierstead R.W. (1958). The total synthesis of reserpine. Tetrahedron.

[29] Bailey P.D., Cochrane P.J., Lorenz K., Collier I.D., Pearson D.P.J., Rosair G.M. (2001). A concise, efficient route to fumitremorgins. Tetrahedron Lett..

[30] Koskinen A.M.P. (2012). Asymmetric Synthesis of Natural Products.

[31] Bailey P.D., Hollinshead S.P., McLay N.R., Morgan K., Palmer S.J., Prince S.N., Reynolds C.D., Wood S.D. (1993). Diastereo- and enantio-selectivity in the pictet-spengler reaction. J. Chem. Soc. Perkin Trans. 1.

[32] Ardeo A., García E., Arrasate S., Lete E., Sotomayor N. (2003). A practical approach to the fused β-carboline system. asymmetric synthesis of indolo[2,3-a]indolizidinones via a diastereoselective intramolecular α-amidoalkylation reaction. Tetrahedron Lett..

[33] Li J., Wang T., Yu P., Peterson A., Weber R., Soerens D., Grubisha D., Bennett D., Cook J.M. (1999). General approach for the synthesis of ajmaline/sarpagine indole alkaloids: Enantiospecific total synthesis of (+)-ajmaline, alkaloid g, and norsuaveoline via the asymmetric pictet-spengler reaction. J. Am. Chem. Soc..

[34] Liu X., Deschamp J.R., Cook J.M. (2002). Regiospecific, enantiospecific total synthesis of the alkoxy-substituted indole bases, 16-epi-n_a_-methylgardneral, 11-methoxyaffinisine, and 11-methoxymacroline as well as the indole alkaloids alstophylline and macralstonine. Org. Lett..

[35] Bailey P.D., Clingan P.D., Mills T.J., Price R.A., Pritchard R.G. (2003). Total synthesis of (−)-raumacline. Chem. Commun..

[36] Alberch L., Bailey P.D., Clingan P.D., Mills T.J., Price R.A., Pritchard R.G. (2004). The cis-specific pictet-spengler reaction. Eur. J. Org. Chem..

[37] Bailey P.D., Beard M.A., Phillips T.R. (2009). Unexpected cis selectivity in the pictet–spengler reaction. Tetrahedron Lett..

[38] Yamada H., Kawate T., Nishida A., Nakagawa M. (1999). Asymmetric addition of alkyllithium to chiral imines: α-naphthylethyl group as a chiral auxiliary. J. Org. Chem..

[39] Soe T., Kawate T., Fukui N., Nakagawa M. (1995). Asymmetric pictet-spengler reaction with chiral-n-(β-3-indolyl)ethyl-1-methylbenzylamine. Tetrahedron Lett..

[40] Waldmann H., Schmidt G., Henke H., Burkard M. (1995). Asymmetric pictet-spengler reactions employing n,n-phthaloyl amino acids as chiral auxiliary groups. Angew. Chem. Int. Ed. Engl..

[41] Ducrot P., Rabhi C., Thal C. (2000). Synthesis of tetrahydro-β-carbolines and studies of the pictet–spengler reaction. Tetrahedron.

[42] Kawate T., Yamada H., Soe T., Nakagawa M. (1996). Enantioselective asymmetric pictet-spengler reaction catalyzed by diisopinocampheylchloroborane. Tetrahedron Asymmetry.

[43] Raheem I.T., Thiara P.S., Peterson E.A., Jacobsen E.N. (2007). Enantioselective pictet-spengler-type cyclizations of hydroxylactams: H-bond donor catalysis by anion binding. J. Am. Chem. Soc..

[44] Klausen R.S., Jacobsen E.N. (2009). Weak bronsted acid thiourea co-catalysis: Enantioselective, catalytic protio-pictet-spengler reactions. Org. Lett..

[45] Seayad J., Seayad A.M., List B. (2006). Catalytic asymmetric pictet-spengler reaction. J. Am. Chem. Soc..

[46] Wanner M.J., van der Haas R.N.S., de Cuba K.R., van Maarseveen J.H., Hiemstra H. (2007). Catalytic asymmetric pictet-spengler reactions via sulfenyliminium ions. Angew. Chem..

[47] Priebbenow D.L., Henderson L.C., Pfeffer F.M., Stewart S.G. (2010). Domino Heck-aza-Michael reactions: Efficient access to 1-substituted tetrahydro-β-carbolines. J. Org. Chem..

[48] Rixson J.E., Chaloner T., Heath C.H., Tietze L.F., Stewart S.G. (2012). The development of Domino reactions incorporating the Heck reaction: The formation of N-heterocycles. Eur. J. Org. Chem..

[49] Priebbenow D.L., Stewart S.G., Pfeffer F.M. (2012). Asymmetric induction in domino heck-aza-michael reactions. Tetrahedron Lett..

[50] Meyers A.I., Sohda T., Loewe M.F. (1986). An asymmetric synthesis and absolute configuration of (S)(−)-deplancheine. J. Org. Chem..

[51] Meyers A.I., Miller D.B., White F.H. (1988). Chiral and achiral formamidines in synthesis. The first asymmetric route to (−)-yohimbine and an efficient total synthesis of (±)-yohimbine. J. Am. Chem. Soc..

[52] Gul W., Hamann M.T. (2005). Indole alkaloid marine natural products: An established source of cancer drug leads with considerable promise for the control of parasitic, neurological and other diseases. Life Sci..

[53] World Malaria Report 2012.

[54] Kaufman T.S., Rúveda E.A. (2005). The quest for quinine: Those who won the battles and those won the war. Angew. Chem. Int. Ed..

[55] Sidhu A.B.S., Verdier-Pinard D., Fidock D.A. (2002). Chloroquine resistance in *Plasmodium falciparum* malaria parasites conferred by Pfcrt mutations. Science.

[56] Staerk D., Lemmich E., Christensen J., Kharazmi A., Olsen C.E., Jaroszewski J.W. (2000). Leishmanicidal, Antiplasmodial and cytotoxic activity of indole alkaloids from *Corynanthe. pachyceras*. Planta Med..

[57] Kam T., Sim K., Koyano T., Komiyama K. (1999). Leishmanicidal alkaloids from *Kopsia. griffithii*. Phytochemistry.

[58] Chauhan S.S., Gupta L., Mittal M., Vishwakarma P., Gupta S., Chauhan P.M.S. (2010). Synthesis and Biological evaluation of indolyl glyoxylamides as a new class of antileishmanial agents. Bioorg. Med. Chem. Lett..

[59] Kumar A., Katiyar S.B., Gupta S., Chauhan P.M.S. (2006). Syntheses of new substituted triazino tetrahydroisoquinolines and β-carbolines as novel antileishmanial agents. Eur. J. Med. Chem..

[60] Kumar R., Khan S., Verma A., Srivastava S., Viswakarma P., Gupta S., Meena S., Singh N., Sarkar J., Chauhan P.M.S. (2010). Synthesis of 2-(Pyrimidin-2-yl)-1-phenyl-2,3,4,9-tetrahydro-1H-β-carbolines as antileishmanial agents. Eur. J. Med. Chem..

[61] Düsman Tonin L.T., Barbosa V.A., Bocca C.C., Ramos É.R.F., Nakamura C.V., Ferreira da Costa W., Basso E.A., Nakamura T.U., Sarragiotto M.H. (2009). Comparative study of the trypanocidal activity of the methyl 1-nitrophenyl-1,2,3,4-9*H*-tetrahydro-β-carboline-3-carboxylate derivatives and benznidazole using theoretical calculations and cyclic voltammetry. Eur. J. Med. Chem..

[62] Valdez R.H., Tonin L.T.D., Ueda-Nakamura T., Silva S.O., Dias Filho B.P., Kaneshima E.N., Yamada-Ogatta S.F., Yamauchi L.M., Sarragiotto M.H., Nakamura C.V. (2012). *In Vitro* and *in vivo* Trypanocidal synergistic activity of n-butyl-1-(4-dimethylamino)phenyl-1,2,3,4-tetrahydro-β-carboline-3-carboxamide associated with benznidazole. Antimicrob. Agent. Chemother..

[63] Lake R.J., Blunt J.W., Munro M. (1989). Eudistomins from the New Zealand ascidian *Ritterella. sigillinoides*. Aust. J. Chem..

[64] Gudmundsson K., Miller J.F., Sherrill R.G., Turner E.M. (2006). Useful Compounds for HPV Infection.

[65] Kobayashi J., Cheng J.F., Ohta T., Nozoe S., Ohizumi Y., Sasaki T. (1990). Eudistomidins B, C, and D: Novel antileukemic alkaloids from the okinawan marine tunicate *Eudistoma glaucus*. J. Org. Chem..

[66] Adesanya S.A., Chbani M., Paîs M., Debitus C. (1992). Brominated beta-carbolines from the marine tunicate *Eudistoma album*. J. Nat. Prod..

[67] Shen Y., Chen Y., Hsieh P., Duh C., Lin Y., Ko C. (2005). The preparation and evaluation of 1-substituted 1,2,3,4-tetrahydro- and 3,4-dihydro-β-carboline derivatives as potential antitumor agents. Chem. Pharm. Bull..

[68] Santos L.S., Theoduloz C., Pilli R.A., Rodriguez J. (2009). Antiproliferative activity of arborescidine alkaloids and derivatives. Eur. J. Med. Chem..

[69] Kumar R., Gupta L., Pal P., Khan S., Singh N., Katiyar S.B., Meena S., Sarkar J., Sinha S., Kanaujiya J.K. (2010). Synthesis and cytotoxicity evaluation of (tetrahydro-β-carboline)-1,3,5-triazine hybrids as anticancer agents. Eur. J. Med. Chem..

[70] Zeng Y., Zhang Y., Weng Q., Hu M., Zhong G. (2010). Cytotoxic and insecticidal activities of derivatives of harmine, a natural insecticidal component isolated from *Peganum harmala*. Molecules.

[71] Sarli V., Giannis A. (2008). Targeting the kinesin spindle protein: Basic principles and clinical implications. Clin. Cancer Res..

[72] Jackson J.R., Patrick D.R., Dar M.M., Huang P.S. (2007). Targeted anti-mitotic therapies: Can we improve on tubulin agents?. Nat. Rev. Cancer.

[73] Sunder-Plassmann N., Sarli V., Gartner M., Utz M., Seiler J., Huemmer S., Mayer T.U., Surrey T., Giannis A. (2005). Synthesis and biological evaluation of new tetrahydro-β-carbolines as inhibitors of the mitotic kinesin eg5. Bioorg. Med. Chem..

[74] Vennemann M., Braunger J., Gimmnich P., Baer T. (2009). Indolopyridines as Inhibitors of the Kinesin Spindle Protein (Eg5).

[75] 4SC: Product pipeline/SC4–205.

[76] Rath O., Kozielski F. (2012). Kinesins and cancer. Nat. Rev. Cancer.

[77] Liu F., Yu L., Jiang C., Yang L., Wu W., You Q. (2010). Discovery of Tetrahydro-Β-Carbolines as Inhibitors of the Mitotic Kinesin KSP. Bioorg. Med. Chem..

[78] Barsanti P.A., Wang W., Ni Z., Duhl D., Brammeier N., Martin E., Bussiere D., Walter A.O. (2010). The discovery of tetrahydro-β-carbolines as inhibitors of the kinesin Eg5. Bioorg. Med. Chem. Lett..

[79] Schmidt M., Bastians H. (2007). Mitotic drug targets and the development of novel anti-mitotic anticancer drugs. Drug Resist. Updat..

[80] Chan K., Koh C., Li H. (2012). Mitosis-targeted anti-cancer therapies: Where they stand. Cell. Death Dis..

[81] Sandeep G., Bhasker S., Ranganath Y.S. (2009). Phosphodiesterase as a novel target in cancer chemotherapy. Internet J. Pharmacol..

[82] El-Gamil D.S., Ahmed N.S., Gary B.D., Piazza G.A., Engel M., Hartmann R.W., Abadi A.H. (2013). Design of novel β-carboline derivatives with pendant 5-bromothienyl and their evaluation as phosphodiesterase-5 inhibitors. Arch. Pharm..

[83] Abadi A.H., Lehmann J., Piazza G.A., Abdel-Halim M., Ali M.S.M. (2011). Synthesis, molecular modeling, and biological evaluation of novel tetrahydro-β-carboline hydantoin and tetrahydro-β-carboline thiohydantoin derivatives as phosphodiesterase 5 inhibitors. Int. J. Med. Chem..

[84] Mohamed H.A., Girgis N.M.R., Wilcken R., Bauer M.R., Tinsley H.N., Gary B.D., Piazza G.A., Boeckler F.M., Abadi A.H. (2011). Synthesis and molecular modeling of novel tetrahydro-β-carboline derivatives with phosphodiesterase 5 inhibitory and anticancer properties. J. Med. Chem..

[85] Ahmed N.S., Gary B.D., Tinsley H.N., Piazza G.A., Laufer S., Abadi A.H. (2011). Design, synthesis and structure-activity relationship of functionalized tetrahydro-β-carboline derivatives as novel PDE5 inhibitors. Arch. Pharm..

[86] Abadi A.H., Piazza G. (2011). Tetrahydro-β-Carboline Derivatives, Synthesis and Use Thereof.

[87] Saxton J.E. (1994). Monoterpenoid Indole Alkaloids: Supplement to Part. 4..

[88] Audia J.E., Evrard D.A., Murdoch G.R., Droste J.J., Nissen J.S., Schenck K.W., Fludzinski P., Lucaites V.L., Nelson D.L., Cohen M.L. (1996). Potent, selective tetrahydro-β-carboline antagonists of the serotonin 2B (5HT2B) contractile receptor in the rat stomach fundus. J. Med. Chem..

[89] Giorgioni G., Accorroni B., Stefano A., Marucci G., Siniscalchi A., Claudi F. (2005). Benzimidazole, Benzoxazole and benzothiazole derivatives as 5ht2b receptor ligands. synthesis and preliminary pharmacological evaluation. Med. Chem. Res..

[90] Glennon R.A., Grella B., Tyacke R.J., Lau A., Westaway J., Hudson A.L. (2004). Binding of β-carbolines at imidazoline I2 receptors: A structure–affinity investigation. Bioorg. Med. Chem. Lett..

[91] Poitout L., Roubert P., Contour-Galcera M., Moinet C., Lannoy J., Pommier J., Plas P., Bigg D., Thurieau C. (2001). Identification of potent non-peptide somatostatin antagonists with Sst3 selectivity. J. Med. Chem..

[92] Pasternak A., Feng Z., de Jesus R., Ye Z., He S., Dobbelaar P., Bradley S.A., Chicchi G.G., Tsao K., Trusca D. (2012). Stimulation of glucose-dependent insulin secretion by a potent, selective Sst3 antagonist. ACS Med. Chem. Lett..

[93] Ganong W. (2005). Review of Medical Physiology.

[94] Zhou Y., Li J., Wu W., Shang J., Thompson J.R., Thornberry N.A. (2009). Diagnosis and Treatment of Type 2 Diabetes and Other Disorders. U.S. Patent.

[95] Mjalli A.M.M., Andrews R.C., Xie R., Yarragunta R.R., Ren T. (2003). Beta-Carboline Derivatives as PTP-Inhibitors. WO03033496A1.

[96] Brandt P., Bremberg U., Crossley R., Graffner Nordberg M., Jenmalm Jensen A., Ringberg E., Ward T. (2005). Novel Beta-Carbolines as Growth Hormone Secretagogue Receptor (GHSR) Antagonists. WO2005048916A2.

[97] Hung D., Protter A., Chakravarty S., Jain R., Dugar S. (2009). Pyrido[3,4-B]indoles and methods of use. WO2009120717.

[98] Ortar G., Petrocellis L.D., Moriello A.S., Allarà M., Morera E., Nalli M., Marzo V.D. (2013). Tetrahydro-β-Carboline derivatives targeting fatty acid amide hydrolase (FAAH) and transient receptor potential (TRP) channels. Bioorg. Med. Chem. Lett..

[99] Bi W., Bi L., Cai J., Liu S., Peng S., Fischer N.O., Tok J.B.-H., Wang G. (2006). Dual-acting agents that possess free radical scavenging and antithrombotic activities: Design, Synthesis, and evaluation of phenolic tetrahydro-β-carboline RGD peptide conjugates. Bioorg. Med. Chem. Lett..

[100] Bi W., Cai J., Liu S., Baudy-Floc’h M., Bi L. (2007). Design, Synthesis and cardioprotective effect of a new class of dual-acting agents: Phenolic tetrahydro-β-carboline RGD peptidomimetic conjugates. Bioorg. Med. Chem..

